# Ion Transport in Polymer Electrolytes: Building New Bridges between Experiment and Molecular Simulation

**DOI:** 10.1021/acs.accounts.3c00791

**Published:** 2024-04-03

**Authors:** Yunqi Shao, Harish Gudla, Jonas Mindemark, Daniel Brandell, Chao Zhang

**Affiliations:** Department of Chemistry—Ångström Laboratory, Uppsala University, Lägerhyddsvägen 1, Box 538, 751 21 Uppsala, Sweden

## Abstract

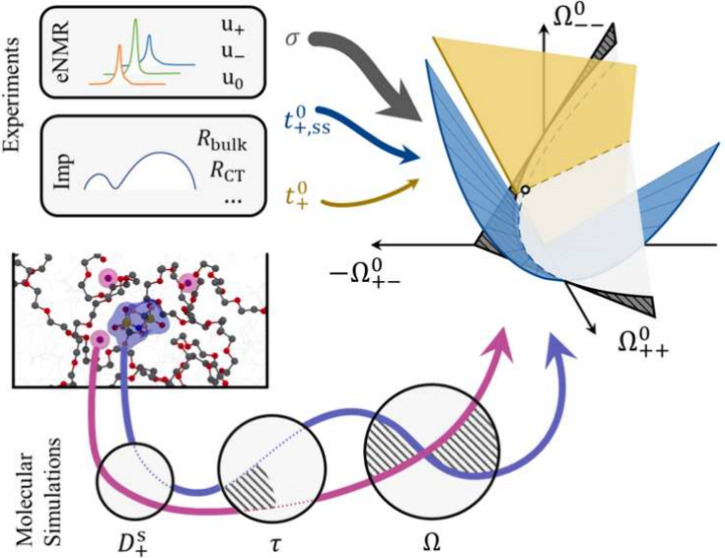

Polymer electrolytes constitute a promising type of material for solid-state batteries. However, one of the bottlenecks for their practical implementation lies in the transport properties, often including restricted Li^+^ self-diffusion and conductivity and low cationic transference numbers. This calls for a molecular understanding of ion transport in polymer electrolytes in which molecular dynamics (MD) simulation can provide both new physical insights and quantitative predictions. Although efforts have been made in this area and qualitative pictures have emerged, direct and quantitative comparisons between experiment and simulation remain challenging because of the lack of a unified theoretical framework to connect them.

In our work, we show that by computing the glass transition temperature (*T*_g_) of the model system and using the normalized inverse temperature 1000/(*T* – *T*_g_ + 50), the Li^+^ self-diffusion coefficient can be compared quantitatively between MD simulations and experiments. This allows us to disentangle the effects of *T*_g_ and the polymer dielectric environment on ion conduction in polymer electrolytes, giving rise to the identification of an optimal solvating environment for fast ion conduction.

Unlike Li^+^ self-diffusion coefficients and ionic conductivity, the transference number, which describes the fraction of current carried by Li^+^ ions, depends on the boundary conditions or the reference frame (RF). This creates a non-negligible gap when comparing experiment and simulation because the fluxes in the experimental measurements and in the linear response theory used in MD simulation are defined in different RFs. We show that by employing the Onsager theory of ion transport and applying a proper RF transformation, a much better agreement between experiment and simulation can be achieved for the PEO–LiTFSI system. This further allows us to derive the theoretical expression for the Bruce–Vincent transference number in terms of the Onsager coefficients and make a direct comparison to experiments. Since the Bruce–Vincent method is widely used to extract transference numbers from experimental data, this opens the door to calibrating MD simulations via reproducing the Bruce–Vincent transference number and using MD simulations to predict the true transference number.

In addition, we also address several open questions here such as the time-scale effects on the ion-pairing phenomenon, the consistency check between different types of experiments, the need for more accurate force fields used in MD simulations, and the extension to multicomponent systems. Overall, this Account focuses on building new bridges between experiment and simulation for quantitative comparison, warnings of pitfalls when comparing apples and oranges, and clarifying misconceptions. From a physical chemistry point of view, it connects to concentrated solution theory and provides a unified theoretical framework that can maximize the power of MD simulations. Therefore, this Account will be useful for the electrochemical energy storage community at large and set examples of how to approach experiments from theory and simulation (and vice versa).

## Key References

- GudlaH; ZhangC.; BrandellD.Effects of solvent polarity on Li-ion diffusion in polymer electrolytes: An all-atom molecular dynamics study with charge scaling. J. Phys. Chem. B2020, 124, 8124–813132840375 10.1021/acs.jpcb.0c05108PMC7503542.^1^*Focusing on the self-diffusion coefficients, we discussed the importance of including effective temperature and polymer dielectric environment in the quantitative comparison between experiment and simulation.*
- ShaoY.; GudlaH; BrandellD.; ZhangC.Transference number in polymer electrolytes: mind the reference-frame gap. J. Am. Chem. Soc.2022, 144, 7583–758735446043 10.1021/jacs.2c02389PMC9074101.^2^*We discussed the importance of reference frame when comparing the classical (true) transference numbers in experiment and simulation.*
- ShaoY.; ZhangC.Bruce–Vincent transference numbers from molecular dynamics simulations. J. Chem. Phys.2023, 158, 16110437096852 10.1063/5.0146608.^3^*We clarified the definition of the so-called Bruce–Vincent transference number in terms of the Onsager coefficients, which allows a direct comparison between experiment and simulation.*

## 1 Introduction

Poly(ethylene oxide) (PEO) was the first reported polymer electrolyte with measured ionic conductivity.^4^ Early studies of polymer electrolytes could observe a strong correlation between polymer morphology and conductivity. A special focus was put on the temperature dependence of the conductivity, from which the dynamic bond percolation (DBP) theory was developed and applied in molecular simulation.^5,6^ This captures the unique features of polymers, such as glass transition and segmental motions, and connects them to ionic mobility.

**Figure 1:**
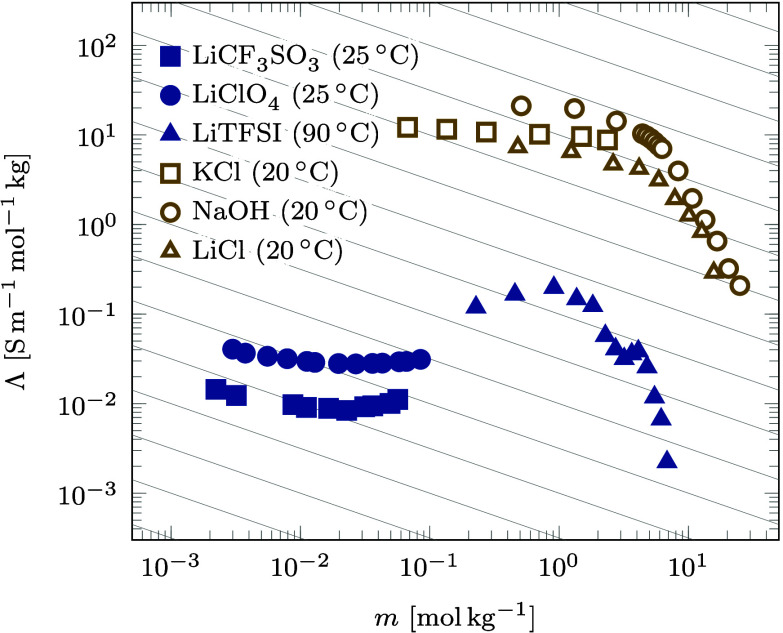
Experimentally measured molal conductivities (Λ) for LiCF_3_SO_3_,^7^ LiClO_4_,^7^ and LiTFSI^8^ in PEO as a function of the salt concentration in logarithm scale. Results for KCl,^9^ NaOH,^10^ and LiCl^11^ in aqueous solutions are also shown to give a perspective. A square-root scaling relation is plotted as solid lines.

In terms of the concentration dependence, classical electrolyte theories based on the Debye–Hückel–Onsager (DHO) approach are valid only for dilute electrolytes below 0.01 mol kg^–1^. For example, the molal conductances of LiCF_3_SO_3_ and LiClO_3_ follow the square-root limiting law^7^ (also see Figure 1). At higher concentrations, normal for practical polymer electrolytes such as the classic PEO–LiTFSI system (Figure 1), the salt-dependent conductivity deviates significantly from the DHO theory, and empirical equations with higher-order fitting parameters no longer contain significant physical meaning.

Thus, a major challenge for studying ion transport in polymer electrolytes and concentrated electrolyte systems alike is how to include ion correlations into our understanding of transport coefficients at higher concentrations. A direct consequence is that approximations based on dilute solution theories are no longer valid, leading to conceptual difficulties in understanding experimental measurements. One such example is transference number measurements, for which different approximations exist that are valid only at low concentration. It was noticed at an early stage that those numbers are not equivalent,^12^ but it is only recently that their significant difference has been shown and more profoundly discussed.^2,3,13,14^

The situation is even more pressing with recent advances in experimental techniques and computational capability, allowing for more accurate determination of the transport properties.^15−18^ Those measurements and simulations can only be understood if the ion transport properties are put into a unified theoretical framework that connects experiment and simulation.

**Figure 2:**
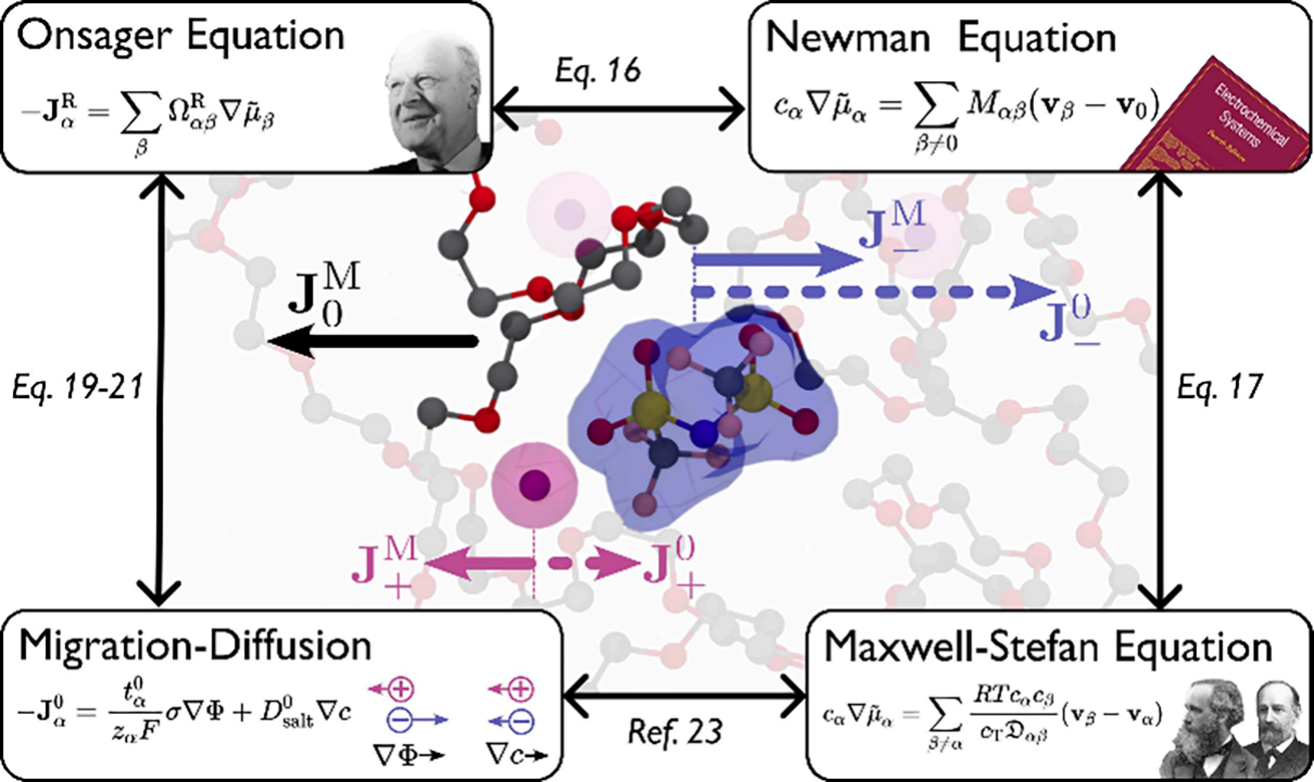
Summary of ion transport theories and their interconversions discussed in section 2. The central panel illustrates the origin of the reference frame dependence, where the cation flux changes the sign when referring to the solvent velocity.

In this Account, we revisit the ion transport in polymer electrolytes by taking the physical chemistry route started by Onsager and discuss our recent progress on bridging experiments and simulation. The text is organized as follows:

First, we introduce the different transport coefficients using Onsager’s framework of ion transport and discuss their reference frame (RF) dependence. This is followed by a description of their evaluations through experiments and simulations and clarification of their relations with respect to other alternative and equivalent theories. The mathematical symbols, if not mentioned in the text, can be found in Table 1. The core of this theoretical section is also summarized in Figure 2 for a skim-through. Readers might find it easier to first read section 3 (also 4) and go back (and forth) to section 2 to check up with formulas.

**Table 1:** List of Symbols and SI Units

symbol	SI unit	description
*z*_α_		charge number
*F*	C mol^–1^	Faraday constant
*R*	J K^–1^ mol^–1^	gas constant
*N*_A_	mol^–1^	Avogadro constant
*a*_α_^R^		weighting factor of R reference frame
*A*_αβ_^RS^		conversion matrix element from R to S
*c*_α_	mol m^–3^	molar concentration of α
μ_α_	J mol^–1^	chemical potential of α
μ̃_α_	J mol^–1^	electrochemical potential of α
ϕ	V	electrostatic potential
Φ	V	migration potential
ω_α_		mass fraction of α
**J**_α_^R^	mol m^–2^ s^–1^	current density of α
**v**_α_	m s^–1^	average velocity of α
Ω_αβ_^R^	mol^2^ J^–1^ m^–1^ s^–1^	Onsager coefficient
	m^2^ s^–1^	Maxwell–Stefan diffusion coefficient
*K*_αβ_	J s m^–5^	Maxwell–Stefan friction coefficient
*M*_αβ_	J s m^–5^	modified Maxwell–Stefan friction coefficient
σ	S m^–1^	ionic conductivity
Λ	S m^2^ mol^–1^	molar conductivity
*t*_α_^R^		transference number of α
*D*_salt_^R^	m^2^ s^–1^	salt diffusion coefficient
*D*_α_^s^	m^2^ s^–1^	self-diffusion coefficient

Next, we discuss different versions of transference numbers under this unified framework and our corresponding works regarding the PEO–LiTFSI system.

Finally, we conclude and give a perspective on several remaining issues with current simulation and experimental techniques and how they may be improved and used for designing new types of polymer electrolytes and studying complex electrolyte systems.

## 2 Theoretical Framework

For the steady-state fluxes in the regime with small electric fields and concentration gradients, a linear relation can be established between the driving force and the response. The relation generally takes the form
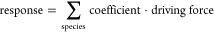
1A list of different instances of such an equation is shown in Table 2.

**Table 2:** Linear Transport Equations Used for Ion Transport Studies in the Literature

equation	response	coefficients	driving force	summation
Onsager (eq 2)	**J**_α_^R^	Ω_αβ_^R^	–∇μ̃_β_	all β
Maxwell–Stefan (eq 14)	*c*_α_∇μ̃_α_		**v**_β_ – **v**_α_	β ≠ α
Newman (eq 15)	*c*_α_∇μ̃_α_	*M*_αβ_	**v**_β_ – **v**_0_	β ≠ 0
migration–diffusion (eq 18)	**J**_α_^0^	σ, *t*_α_^0^, *D*_salt_^0^	–∇Φ, −∇*c*	

It is worth mentioning that these equations are phenomenological in nature, and the corresponding coefficients can be interpreted as either susceptibility or friction. This point is especially important for the present problem, where the fluxes and the driving force are interdependent variables.

On the other hand, since all of these formulations are describing the same phenomenon, the corresponding equations (Table 2) shall have the same degrees of freedom, and the coefficients can be converted between each other exactly.

In the following, we discuss the ion transport based on the Onsager equation. We first introduce the Onsager coefficients and their conversions across different RFs. Then we discuss how the Onsager coefficients connect to other types of transport coefficients, which can be directly obtained from experiments. Finally, we show how the Onsager coefficients in different RFs can be computed from molecular simulation.

### 2.1 Onsager Equation and Coefficients

We consider the phenomena of ion transport in the following form known as the Onsager equation:^19^
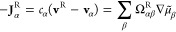
2where **J**_α_^R^ is the flux of species α defined under reference velocity **v**^R^, −∇μ̃_β_ is the driving force acting on species β due to the electrochemical potential, and Ω_αβ_^R^ is the so-called Onsager coefficient.

Before moving on, it is worth commenting on the origin of the RF. The flux is a response property, and therefore, **J**_α_^R^ is not defined without choosing a reference velocity **v**^R^. Since the driving force does not depend on the choice of reference velocity, the corresponding Onsager coefficients will inherit this RF dependence. Moreover, the total current (density), which is ∑_α_*z*_α_*F***J**_α_^R^, turns to be RF-independent because of the charge neutrality.

It is easy to show that such interdependency renders the Onsager coefficients nonunique with eq 2 alone, i.e., a different set of Ω_αβ_^R^ can equally satisfy eq 2. However, constraints follow the Onsager reciprocal relation, and the RF definition can be introduced to alleviate this issue:^20^
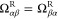
3
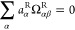
4where *a*_α_^R^ is the weighting factor for a chosen RF.

Equation 4 is related to two linear equations:
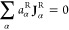
5
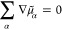
6Equation 5 states the RF under which the current is measured, and eq 6 is the Gibbs–Duhem equation. These make a *n*-component system have only *n* – 1 degrees of freedom.

Given the above constraints in eqs 3 and 4, the Onsager coefficients in a given RF are uniquely defined, and the transformation between different RFs can be shown to be^21,22^
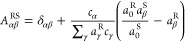
7
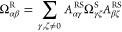
8To make a concrete example, consider a binary 1:1 electrolyte and the transformation from the barycentric RF (M) to the solvent-fixed RF (0). Here we denote the solvent species as “0” and the cation and anion species as “+” and “–”, respectively. Then the *a* factors are
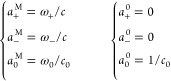
where ω_α_ is the weight fraction of species α and *c* = *c*_+_ = *c*_–_ are the molar concentrations. The transformation factors between the two reference frames are
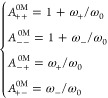


An intuitive understanding of the *A* factors is that they convert fluxes in different RFs using only the cation and anion fluxes. Taking **J**_+_^0^ as an example:
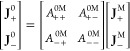
9which can be written explicitly as
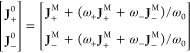
10where one can recognize (ω_+_**J**_+_^M^ + ω_–_**J**_–_^M^)/ω_0_*c* as the negative solvent velocity in the barycentric RF.

Then, using eq 8, we can also convert the Onsager coefficients from a barycentric RF to a solvent-fixed RF or vice versa. The formulas for the 1:1 binary system are
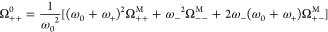
11
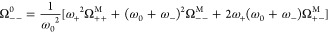
12
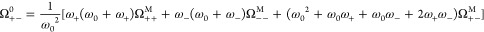
13

The significance of these RF transformations is twofold: (i) Since the default RF in the molecular simulation (see section 2.3) is the barycentric RF, Ω_αβ_^M^ are computed in the MD simulations. However, Ω_αβ_^0^ should be used to determine the classical transference number *t*_+_^0^ (see section 2.2). (ii) Despite the central role of transference number, as highlighted in this Account, it is the complete set of Onsager coefficients that gives the full picture of the ion transport (see section 4 and Figure 8).

### 2.2 Conversion with the Concentrated Solution Theory

Concentrated solution theory, developed by Newman and co-workers, has been instrumental in describing electrolyte systems,^23^ in particular for battery applications. It has been noticed from the start that its formulation is similar to the Stefan–Maxwell equation and equivalent to the Onsager theory of ion transport.^24^ In this section, we will make this connection clear between the Onsager equation and concentrated solution theory as well as other types of flow equations.

The Maxwell–Stefan equation (also see Table 2), popular in the study of multicomponent diffusion,^25^ describes the relation between the driving force and relative velocities between different species:
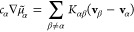
14in which *c*_T_ = ∑_α_*c*_α_ and
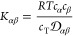
where the latter may be intuitively interpreted as a “friction coefficient” between species α and β. The advantage of this description is that it does not depend on the choice of reference frame. However, experimentally derived Maxwell–Stefan diffusion coefficients  show singularities near certain concentrations.^8^

A similar description was used by Wheeler and Newman,^26^ where the solvent-fixed velocities are used in place of the relative velocities between species:
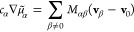
15One can show that eq 15 has straightforward connections to both eq 2 and eq 14:
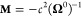
16
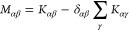
17which gives the general relation for the conversion of transport coefficients between different transport theories.

Another notable form of the flow equations, common in the electrochemistry literature,^23^ is the migration–diffusion equation, where the flows are separated into a migration term due to the electric field and a diffusion term due to the concentration gradient of the salt. The equation is written in the solvent-fixed frame of reference, where the solvent-related terms are zero:
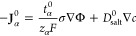
18where *c* is the salt concentration. Equation 18 may be derived from eq 2, with the experimentally measurable ionic conductivity σ and classical transference number *t*_α_^0^:
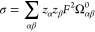
19
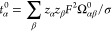
20For a single-salt system, the salt diffusion coefficient can also be derived:
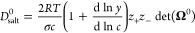
21where () is the thermodynamic factor in molar units and *y* is the corresponding activity coefficient.

Here, the potential Φ in eq 18 must be distinguished from the electrostatic potential ϕ as follows:
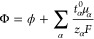
22The potential Φ is equivalent only to the electrostatic potential ϕ at uniform concentration (∇*c* = 0).

The significance of eq 18 is its direct correspondence with experimental measurements. Under ∇*c* = 0, σ and *t*_α_^0^ correspond to the ionic conductivity and the classical transference numbers, respectively. ∇Φ = 0 corresponds to the measurement of *D*_salt_^0^ in a concentration cell, when ϕ is equal to the diffusion potential.

### 2.3 Time Correlation Functions and Molecular Simulations

The transport theories introduced so far are phenomenological theories, which are complete and consistent without assuming the underlying molecular nature of the system.^27,28^

To connect the phenomenological transport coefficients and microscopic entities, one would need to resort to statistical mechanics. Through this critical step, we can then compute these transport coefficients from the computational version of statistical mechanics, i.e., MD simulations.

Central to this development is Onsager’s regression hypothesis, which assumes that the relaxation of macroscopic nonequilibrium disturbances is governed by the same laws as the regression of spontaneous microscopic fluctuations in an equilibrium system.^29^ This leads to the development of various versions of the Green–Kubo relation, which provides the necessary bridges.^30^

The most familiar example of this kind is the expression for computing the self-diffusion coefficient (*D*_α_^s^):
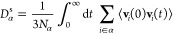
23where **v**_*i*_ is the velocity of the labeled atom *i* and *N*_α_ is the number of α particles in the system. The self-diffusion coefficient is estimated by averaging over all particles belonging to species α. The equivalent Einstein equation for *D*_α_^s^ is then
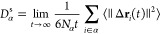
24in which we use the displacement of each atom Δ**r**_*i*_ instead.

One can see self-diffusion as a special case of eq 2 where the concentration of the labeled particle is infinitely low.^31^ Therefore, its activity coefficient is 1 by definition, and the driving force is −∇μ_α_ = −*RT*∇(ln *c*_α_) for the labeled species α. This recovers Fick’s first law:
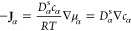
25In this case, we can also drop the RF dependence since all RFs are the same when the only mobile species is infinitely dilute. This leads further to the Nernst–Einstein relation for ionic conductivity:
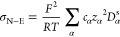
26

Following the same line of thought, the Green–Kubo relation for the Onsager coefficient Ω_αβ_^R^ reads
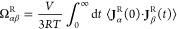
27where the system has a volume of *V* and ⟨···⟩ here denotes the ensemble average over different trajectories and particles belonging to the same species. This expression can also be written in the form of the Einstein relation:
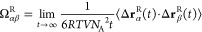
28where Δ**r**_α_^R^(*t*) is the total displacement of α within the volume over the time span *t*.

It is worth noting that the RF transformation can be understood in terms of the transformation of RF under which the correlation functions are computed, namely:
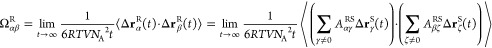
29which reduces to eq 8. This means that the relation between transport coefficients and time correlation functions holds under any reference frame. Thus, a consistent interpretation of the RF dependence of Ω_αβ_^R^ should consider the transformation of correlation functions. In particular, as mentioned before, the default reference frame used in the MD simulations is the barycentric RF, and the direct application of eq 27 leads to Ω_αβ_^M^. By performing the RF transformation listed above, one can make the connection to concentrated solution theory and the corresponding measurements via Ω_αβ_^0^.

Finally, it is worth noting that the derivation of Maxwell–Stefan diffusion coefficients  from time correlation functions was only verified in two-component systems^26^ and the most rigorous pathway to obtain Maxwell–Stefan diffusion coefficients in higher-component systems is again through the corresponding Onsager coefficients^32^ (also see eqs 16 and 17).

## 3 Transference Numbers

The elaborated introduction in the previous sections paves the way to discuss a key property in polymer electrolytes, the transference number *t*_+_, which is directly linked to the limiting current of the battery cell with an interelectrode distance of *L*:^33^
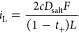
30There are different types of transference numbers commonly reported in the literature,^16^ as defined in Table 3. They differ from each other by the degree of ion correlations included in the expression and converge to the same quantity at the limit of an infinitely dilute solution, i.e. Ω_+–_^0^ → 0, Ω_++_^0^ → *D*_+_^s^, and Ω_––_^0^ → *D*_–_^s^. It is worth noting that the quantities listed Table 3 are different from the transference number of solvated ions,^27,28^ i.e., the transport number. Although intuitive and conventional following the pioneering work of Hittorf, the latter introduced an assumption regarding the speciation (free ions versus ion pairs) in the system.

**Table 3:** List of Commonly Reported Transference Numbers and Their Expressions (for 1:1 Binary Electrolytes), RF Dependences, Experimental Techniques, and Availability^a^

quantity	expression	RF-dependent?	experiments	availability
*t*_+,app_	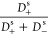	no	PFG-NMR	medium
*t*_+_^0^	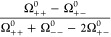	yes	eNMR, Newman method	low
*t*_+,ss_	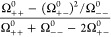	yes	Bruce–Vincent, VLF-Imp.	high

a Abbreviations: PFG, pulsed-field gradient; eNMR, electrophoretic NMR; VLF-Imp., very-low-frequency impedance spectroscopy.

In addition to keeping in mind how ion correlations are treated in each case, another important point to realize is the RF dependence of transference numbers. Therefore, the aim of this section is to showcase our work on comparing different types of transference numbers for the PEO–LiTFSI system between experiments and simulations by considering both of these aspects.

### 3.1 Apparent Transference Number *t*_+,app_

The self-diffusion coefficients (*D*_α_^s^) characterize the mobility of the individual ions. While they are not directly linked to the phenomena of ion transport described in eq 2, they are more attainable from both experiments and simulations. *D*_α_^s^ values do not involve the complication of RF transformation and boundary conditions, which makes them favorable choices when comparing experimental data and simulations.

*D*^s^ can be used as a first indicator of how the polymer–ion interaction affects the transport mechanism. This is best demonstrated by the temperature dependence of the ionic conductivity and diffusion coefficient. In polymer electrolytes, ion transport is characterized by the Vogel–Fulcher–Tammann (VFT) relation because of the coupling to the segmental motion of polymer chains.^34^ This comes in contrast to the case of ion transport via hopping between coordination sites in crystalline materials, as characterized by the Arrhenius relation.^35^

**Figure 3:**
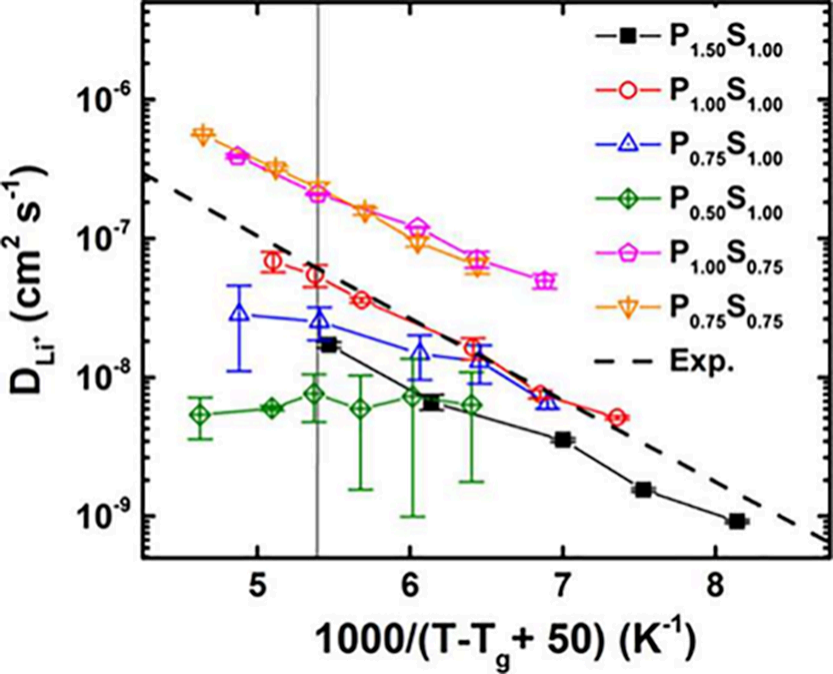
Li^+^ self-diffusion coefficient as a function of the normalized inverse temperature. The vertical line corresponds to 1000/(*T* – *T*_g_ + 50) = 5.4 (±0.1). Each simulation system is labeled according to the scaling factor *f* on the point charges in a polymer (P) and salt (S). Reproduced from ref (1). Copyright 2020 American Chemical Society.

Thus, it is important that the comparison between MD simulations and experiments is performed on an appropriate temperature scale with a VFT-type expression. We showed in a previous study that a good agreement on the temperature-dependent self-diffusion coefficient can be achieved if a normalized inverse temperature 1000/(*T* – *T*_g_ + 50), based on the glass transition temperature (*T*_g_), is used, as shown in Figure 3.

The choice of temperature scale is important for two reasons: (1) The *T*_g_ of simulation systems is often noticeably different from experimental values due to limitations of the force field parameters. It is only through a normalized temperature dependence that a match between transport mechanisms can be ascertained. (2) The effects of *T*_g_ and the polymer dielectric environment are intricately entangled.^36^ By taking the same effective temperature, *T* – *T*_g_, one can disentangle these effects.

**Figure 4:**
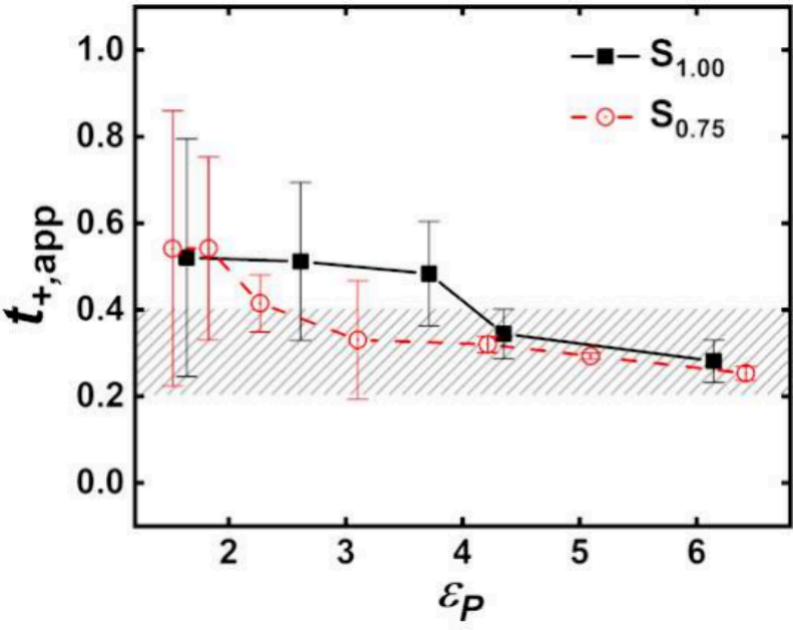
Apparent transference number as a function of the solvent polarity at the normalized inverse temperature 1000/(*T* – *T*_g_ + 50) = 5.4 ± 0.1. The gray region corresponds to the expected values. Adapted from ref (1). Copyright 2020 American Chemical Society.

As demonstrated in Figure 3, the effect of charge scaling schemes in the force field is revealed when comparing *D*_+_^s^ to the normalized inverse temperature scale, showcasing the influence of polymer polarity on the transport mechanism. At the same normalized inverse temperature (the vertical line in Figure 3), it is found that *t*_+,app_ is systematically correlated with the solvent polarity, as shown in Figure 4.

Therefore, determining *T*_g_ and using the effective temperature *T* – *T*_g_ is a recommended practice when comparing experiments and simulations of polymer electrolytes. On the other hand, the determination of *T*_g_ in the simulation system can also be tricky depending the simulation protocol (e.g., the cooling rate). We are in the process of writing a separate comment regarding this point, which is worth a detailed technical discussion on its own.

### 3.2 Classical Transference Number *t*_+_^0^

Despite the usefulness of self-diffusion data for aligning simulation with experiments, *t*_+,app_ almost never agrees with the transference number *t*_+_^0^ defined by “classical” measurements such as the Hittorf method or moving boundary methods,^37^ which are difficult to set up for polymer systems.^12^ The difficulty is addressed by the Newman approach by combining a set of electrochemical measurements^38^ and more recently by the application of electrophoretic NMR (eNMR) measurements^39^ to polymer electrolytes.

**Figure 5:**
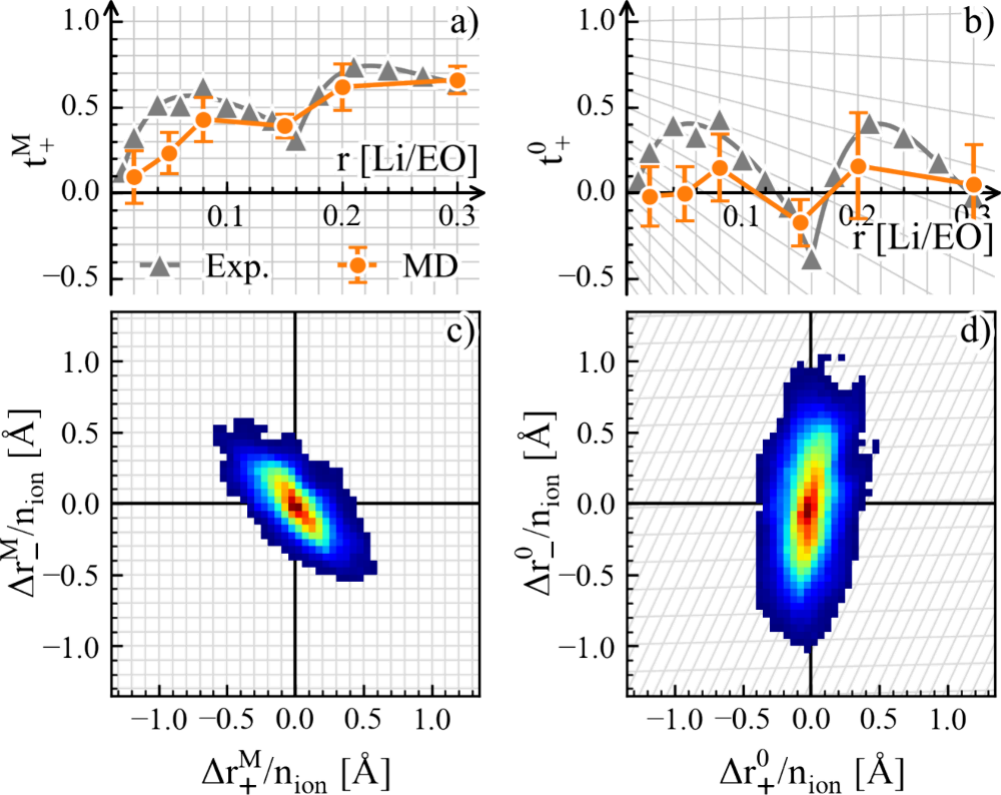
RF transformation of (a, b) transference number measured with the Newman method^8^ and MD simulations and (c, d) the cross-correlation between ion motion visualized as the distribution of ion displacement within a given time interval in the MD simulation. Reproduced from ref (2). CC BY 4.0.

A rather interesting and recent observation is that *t*_+_^0^ of PEO–LiTFSI can be negative at concentrations around *r* = 0.17 [Li/EO] when employing the Newman method.^40^ As outlined above, it is possible to estimate *t*_+_^0^ from the Onsager coefficients following eq 20. We showed previously that a similar concentration dependence of *t*_+_^0^ can be obtained in MD simulations^2^ (see Figure 5).

One direct consequence of the RF dependence is that the transference numbers must be compared in the same RF. For estimations from MD simulations, this simply means that the correlation functions should be evaluated in the correct RF or converted to it.

The RF dependence also makes it nonintuitive to interpret results involving charged cluster species, which may be quantified by estimating the cluster population and cluster mobility in MD simulations. These cluster analysis models often assume independent motion between clusters, similar to the Nernst–Einstein relation (eq 26). In light of Figure 5, this assumption can only be valid for one RF. Thus, the critical question to be answered before these cluster analyses can give quantitative predictions of the transport coefficients is under which RF are the clusters moving “independently”. This can be the main reason why systematic deviation is seen in transference number predictions based on cluster population and mobilities.^41^

**Figure 6:**
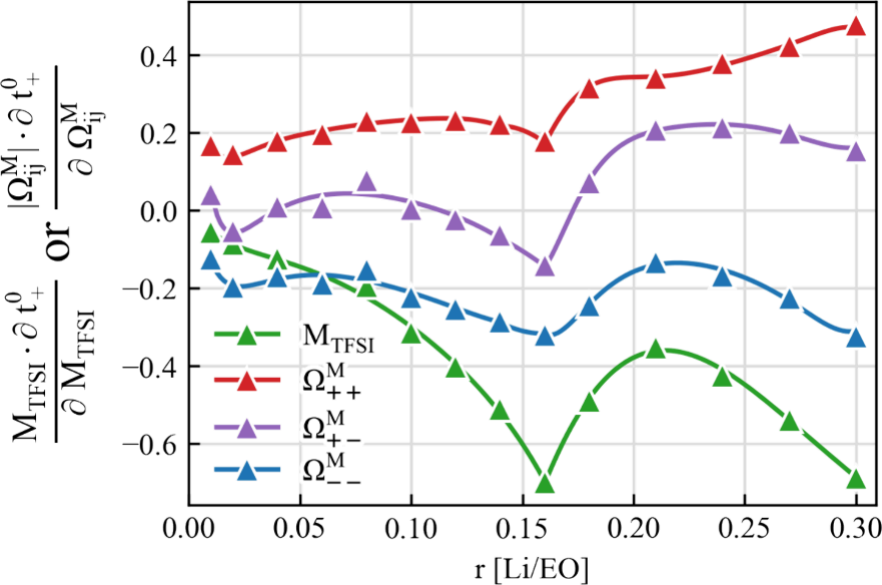
Sensitivity of transference number *t*_+_^0^ in solvent-fixed RF to the variations in the anion molecular weight *M*_TFSI_ and different Onsager coefficients Ω_*ij*_^M^ in the barycentric RF. The analysis is done by evaluating the partial derivative of *t*_+_^0^ with respect to the logarithm of *M*_TFSI_ or |Ω_*ij*_^M^|. Reproduced from ref (2). CC BY 4.0.

On the other hand, the RF dependence also allows us to shed light on the influences of different Onsager coefficients on the classical transference number *t*_+_^0^. As shown in Figure 6, *t*_+_^0^ depends not only on Ω_++_^M^ – Ω_+–_^M^ but also on Ω_––_^M^ and the anion mass fraction. The partial derivative of *t*_0_^+^ (*y* axis in Figure 6) shows a strong dependence on both the anion mass and the anion–anion correlation. An increase in the anion mass introduces a strong reduction of the transference number *t*_0_^+^, and therefore, *t*_0_^+^ is more likely to be negative. The same effect happens when the anion–anion correlation becomes stronger and Ω_––_^M^ becomes larger. This suggests the important role of the anion–anion correlation that comes into play at higher salt concentrations.

### 3.3 Steady-State Transference Number *t*_+,ss_

We close the discussion regarding transference number with the so-called Bruce–Vincent transference numbers. This measurement involves the operation of a cell under anion-blocking conditions, where the steady-state current is measured. This technique and the equivalent Watanabe method are widely applied for polymer electrolytes and concentrated electrolytes,^42−44^ since the elimination of a concentration gradient is more difficult than for liquid electrolytes because of a continuous growth of the diffusion layer.

The Bruce–Vincent transference number is often taken as a crude approximation to the classical transference number *t*_+_^0^, according to the derivation of Bruce and Vincent in the infinite dilution limit.^42^ However, since the measurement will involve the collective motion of ions, the steady-state condition could be described with the Onsager equations at high concentration.

If such an approach is taken, then the steady-state current can be derived from the Onsager coefficients. The essentially same expression was either indicated or implied by Wagner,^45^ Balsara et al.,^46^ and Wohde et al.:^44^
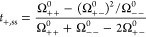
31The remaining discrepancies across those derivations were on (1) the different assumptions regarding the electrostatic and chemical potentials at the steady state and (2) the RF dependence of *t*_+,ss_.

We showed in ref (3) that any such assumption is immaterial since ϕ and μ_+_ cannot be measured independently (the Gibbs–Guggenheim principle), and eq 31 can be derived directly:
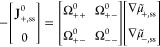
32In addition, we would remark that by comparing eq 32 with eq 2, one should note that eq 32 is only valid under the solvent-fixed RF, since only there are the Ω_0α_^0^ terms zero by definition. In other RFs, the driving force acting on the solvents must also be present in the equations.

**Figure 7:**
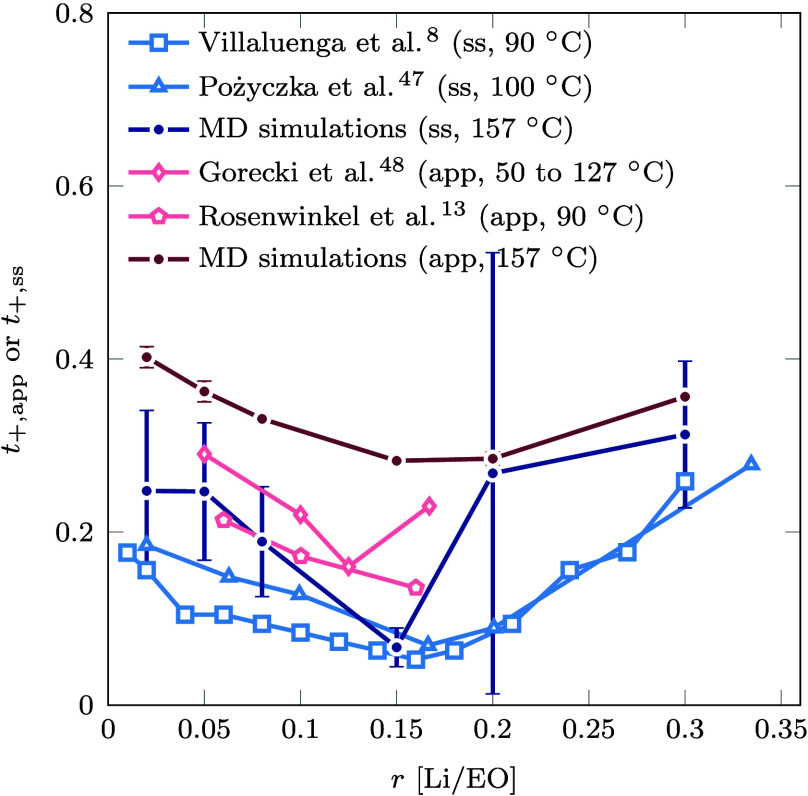
Experimental and simulation results of Bruce–Vincent transference numbers *t*_+,ss_ and apparent transference numbers *t*_+,app_ for the PEO–LiTFSI system. The experimental data were taken from refs (8), (13), (47), and (48). Reproduced from ref (3). CC BY 4.0.

Realizing these points allows us to make a direct comparison of *t*_+,ss_ between experiment and simulation. The results for the PEO–LiTFSI system are listed in Figure 7. It is found that *t*_+,ss_ is always positive, as expected, since both diagonal Onsager coefficients and the determinant are positive; the same is true for *t*_+,app_. The two quantities approach each other under dilute condition.

Before closing this section, we want to make a comment on the relation between *t*_+,ss_ and *t*_+_^0^. In contrast to the common impression, *t*_+,ss_ does contain the ion–ion correlation Ω_+–_^0^. However, this correlation is scaled by a factor of Ω_+–_^0^/Ω_––_^0^ (see Table 3). As revealed in our previous work,^2^ Ω_––_^0^ ≫ Ω_+–_^0^. That is why *t*_+,ss_ is often much larger than *t*_+_^0^.

## 4 Conclusions and Perspectives

Through this Account, we show that the phenomena of ion transport in polymer electrolytes can be described well within the Onsager theory of ion transport and equivalent formulations. With careful examination of the RF choice and boundary conditions, we show that an exact one-to-one comparison for different types of transference numbers between the experimental measurement and molecular simulation is possible.

**Figure 8:**
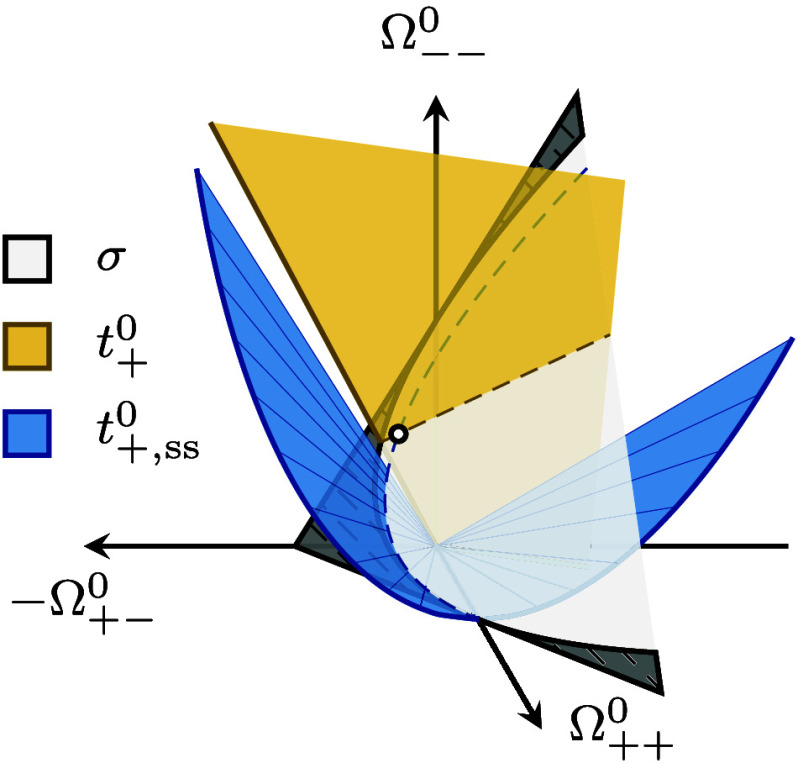
Illustration of the connection between the measured transport coefficients and the Onsager coefficients. Each measurement would correspond to a surface in the space of Ω_αβ_^R^, and a combination of three determines one set of Onsager coefficients.

It is important to realize that neither the conductivity σ nor the transference number (*t*_+,ss_ or *t*_+_^0^) provides complete information about the ion transport. These complementary quantities provide different facets of the same picture, and a combination of them gives a full picture of a binary electrolyte system (Figure 8). This means that only with a set of *N*(*N* – 1)/2 independent transport coefficients, i.e., Onsager coefficients, can one ascertain the correctness of “collective” transport coefficients. In addition, two extra “single-particle” mobilities (*D*_α_^s^) can be supplemented if full agreement is desired.

Despite this progress, there are a number of open questions that remain to be answered when it comes to the ion transport in polymer electrolytes and concentrated electrolyte systems alike. Therefore, in the final section of this Account, we use the theoretical framework described above as a basis for discussing some of these open questions, which call for joint efforts from experimentalists and theoreticians.

### 4.1 Time Scale in Ion-Pairing

Contact ion pairs or ion-pairing is a perennial topic for electrolyte systems. Using transport properties to quantify the extent of ion dissociation (“ionicity”)^49^ can be traced back to Arrhenius, who quantified the dissociation constant with the factor α, which can be expressed as the molar conductivity ratio Λ_c_/Λ_0_, where Λ_0_ is the value at infinite dilution. Moreover, there is spectroscopic evidence showing the existence of contact ion pairs in polymer electrolyte systems.^50^

However, the existence of ion pairs does not equal a negative contribution to the measured ionic conductivity or transference number. This has to do with the fact that ion pairs are usually defined with a thermodynamic criterion, e.g., Bjerrum’s convention, while transport coefficients are dynamical properties. In fact, as we showed previously, Ω_+–_^M^ are mostly negative (meaning cations and anions are anticorrelated) in the entire concentration range^2^ but can become positive depending on the dielectric environment of the polymer matrix.^51^ In the latter case, the time scale of contact ion pairs is long enough to become kinetically relevant to the transport coefficients.

Although it is tempting to attribute any deviation from the Nernst–Einstein relation to ion-pairing, it has already been recognized that this deviation does not necessarily come as the result of a permanent association of ions of opposite charge.^52^ Therefore, one has to be cautious about interpreting results indiscriminately with the concept of ion-pairing. On the other hand, it also provides a great opportunity to explore ion–ion correlations for designing new types of polymer electrolytes, as shown by the long-lived complex between Li^+^ and the ionic liquid cation with oligo(ethylene oxide) side chains^53^ and the balanced polymer–Li^+^ interactions in poly(pentyl malonate) systems.^54^

### 4.2 Experimental Consensus Regarding *t*_+_^0^

A unified theoretical framework for ion transport can be used as a basis for the comparison of different types of experimental measurements. For example, it helps to explain the difference between *t*_+,app_ and *t*_+,ss_ as shown in Figure 7. However, we also notice that a consensus for *t*_+_^0^ has not yet been fully reached.

**Figure 9:**
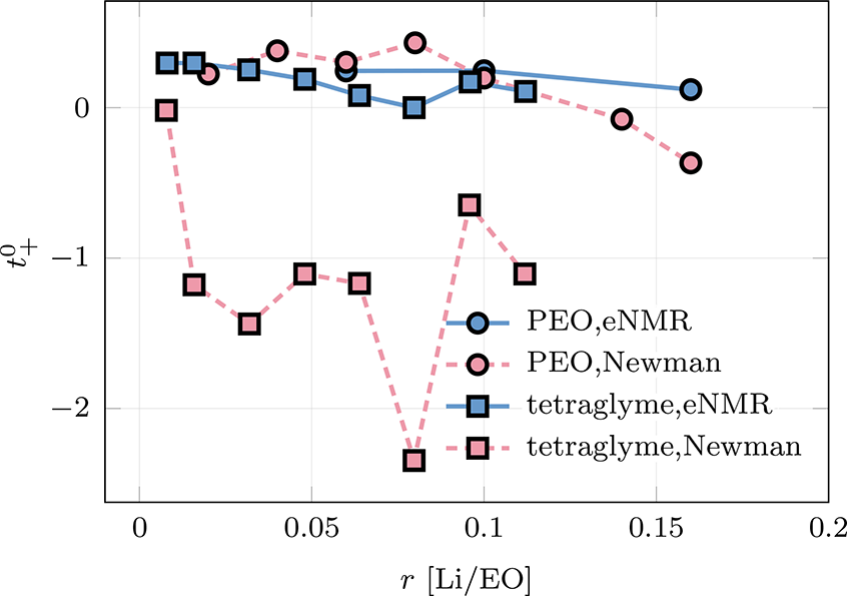
Concentration-dependent transference numbers *t*_+_^0^ from experimental measurements for PEO–LiTFSI^8,13^ and tetraglyme–LiTFSI.^14^

In an eNMR experiment, the measurements can be converted to a specific RF by acquiring the drift velocities of all species. We see that *t*_+_^0^ from eNMR measurements, after conversion to the solvent-fixed RF, is still different from that obtained with the Newman method for the PEO–LiTFSI system (Figure 9). This discrepancy between eNMR measurement and the Newman method becomes apparent for the tetraglyme–LiTFSI system as observed by Hickson et al., who attributed the discrepancy to accumulated errors in the Newman method beyond the simplistic error estimation.^55^ Mistry et al.^14^ examined the condition of eNMR measurements and ruled out the effect of significant concentration gradients within the time scale of eNMR measurements. Therefore, finding a consensus between measurements, after taking into account of the RF dependence,^56^ is still an open quest.

### 4.3 Force Fields Used in MD Simulations

**Figure 10:**
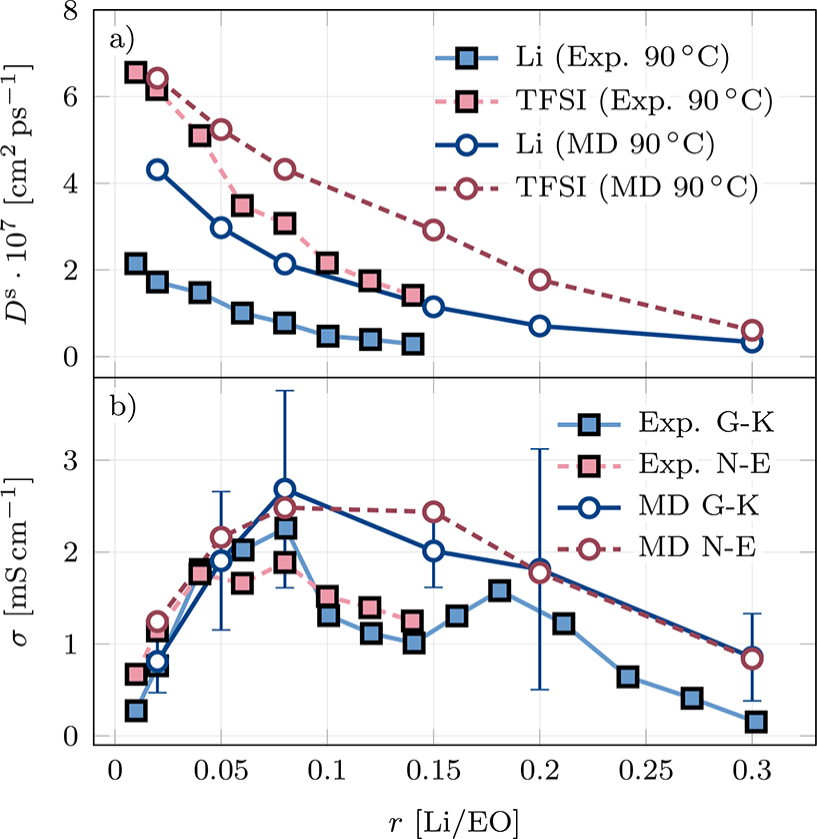
(a) Concentration-dependent self-diffusion coefficients and (b) concentration-dependent ionic conductivities for PEO–LiTFSI from PFG-NMR^40^ and simulations.

In the above discussion, we showed that the force field parameters (*P*_1.00_*S*_0.75_) in ref (2) give a qualitatively correct concentration dependence of the transference number. However, this set of parameters also systematically overestimates the diffusion coefficients, as shown in Figure 10a. The parameter set that gives the best *D*_+_^s^ value in Figure 3 is *P*_1.00_*S*_1.00_ instead.

This illustrates a persisting challenge faced by theoreticians: force fields that can provide accurate predictions for all types of transport coefficients are still lacking. For the development of force fields, in addition to targeting the self-diffusion coefficients, the steady-state transference number *t*_+,ss_ which is highly accessible from experiments, can be used for calibration. Moreover, the thermodynamic factor () can also be computed through the Kirkwood–Buff integral^57^ and benchmarked against experimental measurements. This further allows the salt diffusion coefficient *D*_salt_^0^ to be predicted from molecular simulations using eq 21.

Besides taking the established route of polarizable force fields which include the subtle effect of the electronic polarization,^58^ machine learning potentials (MLPs) that provide the quantum-mechanical accuracy with a fraction of its computational cost constitute an emerging tool for modeling electrolyte systems.^59^ It is likely that their extension from liquid electrolytes to polymer electrolytes will happen in the near future. This would allow us to study both the ion transport and polymer reactivity simultaneously.

### 4.4 Multicomponent Systems

Finally, we note that this discussion focuses on binary electrolyte solutions. Validation of transport coefficients for multicomponent systems, such as those with supporting electrolytes or cosolvents, will face extra complexity in both experimental setup and theoretical derivations.

For instance, the introduction of additional ionic species (e.g., ionic liquids) would lead to additional transference numbers and salt diffusion coefficients in eq 18, whose experimental determination is scarce.

The introduction of additional solvent species means the simplification of results in solvent–ion terms in the Onsager coefficients that cannot be eliminated by the choice of RF. In other words, those terms will enter explicitly in cases where concentration gradients are present (e.g., the Bruce–Vincent transference number).

Nevertheless, these multicomponent systems are of great interest from a practical point of view in electrolyte design,^60^ and the application of the theoretical framework presented in this Account will help us to systematically study these complex and novel electrolyte systems.
